# Supplementary material for: High-volume, label-free imaging for quantifying single-cell dynamics in induced pluripotent stem cell colonies
Source: PLoS One. 2024 Feb 20;19(2):e0298446. doi: 10.1371/journal.pone.0298446 (PMC10878516; doi:10.1371/journal.pone.0298446)
Supplement: S1 Text — (PDF) [file pone.0298446.s001.pdf]

Supplemental Materials

High-volume, label-free imaging for quantifying single-cell dynamics in iPSC colonies

Anthony Asmar[1]*, Zackery A. Benson[1]*, Adele P. Peskin[2], Joe Chalfoun[2], Mylene Simon[2], Michael Halter[1], Anne Plant[1]

1 – Biosystems and Biomaterials Division Material Measurement Lab, NIST Gaithersburg, MD 20899
2 – Software and Systems Division Information Technology Lab, NIST Gaithersburg, MD 20899
*Equal contributors to this work

CONTENTS

1. *Supplemental text*
    a. *Image acquisition (pg. 2-3)*
        i. *Microscope System*
        ii. *General image acquisition workflow*
        iii. *Single time point imaging for collecting image data for training 2D U-Nets*
        iv. *Live cell imaging*
    b. *Imaging instrument benchmarking (pg. 3)*
    c. *Image pre-processing (pg. 3)*
    d. *Considerations for evaluating confidence in AI results (pg. 4)*
    e. *Mean square displacement analysis (pg. 4)*
2. *References (pg. 5)*

Disclaimer: Commercial products are identified in this document in order to specify the experimental procedure adequately. Such identification is not intended to imply recommendation or endorsement by the National Institute of Standards and Technology, nor is it intended to imply that the products identified are necessarily the best available for the purpose.

Image Acquisition

Microscope system

Images were collected with a Zeiss Axio Observer.Z1 microscope (431007-9901-000, Carl Zeiss USA, Thornwood, NY) equipped with motorized x-y stage (MLS203-2, Thorlabs, Newton, NJ) and an ORCA-Fusion BT Digital CMOS camera (C15440-20UP, Hamamatsu, Japan) for image capture. Samples were imaged with phase-contrast illumination using a 590nm LED source (M590L4-C4, Thorlabs, Newton, NJ) set to Kohler conditions corresponding to the Zeiss 10X, 0.3NA objective (420341-9911-000, Carl Zeiss USA) used for imaging. Fluorescence excitation of samples was done using an LED excitation source (pE-4000, CoolLED, Andover, UK) through filter set 38 HE (489038-9901-000, Carl Zeiss USA) for mEGFP imaging and filter set 45 (000000-1114-462, Carl Zeiss USA) for Spy595-DNA imaging. A spatial calibration target was used to determine that each pixel is equivalent to an area of 0.401 μm^2. During automated acquisition, the microscope hardware and x-y stage were controlled with Inscoper software (Inscoper, Rennes, France). Cell experiments were performed with controlled temperature and CO_2 using a microscope incubation chamber (XLmulti S1 DARK LS, PeCon GmBH, Erbach, Germany).

General image acquisition workflow

The general automated acquisition protocol was as follows: 1) A plate focus map was created by scanning a random sample of positions across the imaging area, autofocusing on each position, then estimating the correct focus position for each field of view. 2) The stage moves from field to field to the z-position determined from the plate focus map. Each field overlaps adjacent fields by 10% to facilitate stitching of all fields into a single composite image. At each field, 7 images are acquired above and below the predicted focus position with a z-spacing of 2.5 μm for phase-contrast imaging and a single plane at the predicted focus position for fluorescence.

Single time point imaging for collecting image data for training 2D U-Nets

In this study, we used microscopy instrumentation control that permitted rapid data acquisition, which allowed us to efficiently collect large amounts of image data with a 10x objective for training U-Nets for identification of individual iPSC nuclei. We trained 2D U-Nets with 230,000,000 pixels of image data, which translated up to 215,000 cell/nucleus objects. Approximately 0.2 hours of imaging were required to collect the cell data used for training this 2D U-Net, compared to approximately 6 hours of imaging required for our previous study (1). We automated the annotation of these large training data sets using traditional algorithms to segment fluorescent nuclei, which provided much more training data than would have been available by manual annotation.

Live cell imaging

For live cell experiments, paired phase-only and phase + fluorescence excitation imaging was done for each condition with triplicate wells. Exposure times were kept consistent between experiments with phase-contrast imaging at 100ms and fluorescence excitation at 1000ms. Fluorescence excitation intensity was varied for the different dosages. The imaging sequence involved acquiring a 2x2 tile in each well of a 6-well plate in phase-contrast or both phase-contrast and fluorescence every 2 minutes for 20+ hours. This results in a minimum of 100,000 images to be processed and analyzed per well. All data is written locally before being actively transferred to a larger network attached storage for downstream processing and analysis.

Imaging instrument benchmarking

Periodically (approximately once per month), we performed a series of benchmarking measurements to evaluate the operation of the microscopy system. Incident illumination power at the sample level was measured using a digital optical power meter and slide photodiode power sensor (PM100D and S170C, Thorlabs, Newton, NJ). Testing of stage functionality and repeatability, field distortion, field uniformity, lateral co-registration, and z-stack drift was performed by imaging with the Argo-LM slide and analyzed using the Daybook 3 software (Argolight, Pessac, France). Optical spatial calibration was completed using a cross micrometer (60210-13PG, Electron Microscopy Sciences, Hatfield, PA). A positive 1951 USAF resolution test target (R3L3S1P, Thorlabs) was used to confirm optical resolution. Camera alignment with the optical system was completed using a line grid target (62-536, Edmund Optics, Barrington, NJ).

Image pre-processing

For each time point, the microscope takes multiple field of view images (2000x2000 pixels) over a large, multi-field of view area with 10% overlap between fields of view (also referred to as tiles). Each field of view contains 7 phase-contrast images at varying z planes and a single fluorescence image. Before stitching, the most-in-focus phase image is obtained by selecting the sharpest image, as determined as the z-position with the highest average image derivative computed using Roberts operator. Then, the phase-contrast images are stitched using MIST(2). Fluorescence images are stitched using the same stitching vectors obtained from the phase-contrast channel. Stitching vectors are smoothed across time to avoid jumps that could be due to artefacts like floating debris.

Comparison of model results with different imaging systems

The image data collected for comparing two nominally different microscope systems was collected by imaging a location within a well of iPS cells, then imaging the same well on another microscope. No attempt was made to assure that the imaged locations were identical. Phase contrast and corresponding GFP fluorescence images were acquired as described in the General image acquisition workflow section above. Phase contrast images were inferenced and nuclear fluorescence images were segmented as previously described. The resulting mask images were used to compute F1-scores for each of the microscope systems (n=4,585 and n=5,300 cells for Microscope 1 and Microscope 2, respectively).

The components of each microscope system are listed in the following table. The components of each microscope system were different but may have been the same product from the same vendor (i.e. no component was moved from one system to the other during this evaluation). The similarity of the F1-scores resulting from the analyses provides confidence that image data from the different microscope systems can be combined into a single analysis when quantitatively characterizing cell lines for a research study.

Component	Microscope 1	Microscope 2
**Objective lens**	Zeiss 10X, 0.3NA objective (431007-9901-000)	Zeiss 10X, 0.3NA objective (431007-9901-000)
**Microscope stand**	Zeiss Axio Observer.Z1	Zeiss Axio Observer.Z1
**Digital camera**	ORCA-Fusion BT Digital CMOS camera	ORCA-Fusion BT Digital CMOS camera
**Stage**	Thorlabs MLS203-2	Marzhauser SCANplus IM 130 x 100 (S2400224)
**Transmitted light source**	Thorlabs 590nm LED source (M590L4-C4)	Marzhauser LED 100 (white light)
**Transmitted light condenser**	Zeiss manual condenser (000000-1005-847)	Zeiss motorized condenser (424244-9000-000)
**Fluorescence excitation light source**	CoolLED pE-4000 (470 nm)	Thorlabs 4-Wavelength High-Power LED Source (LED4D118, 470 nm)

**Fluorescence excitation/emission filters**	Zeiss filter set 38 HE (489038-9901-000)	Zeiss filter set 38 HE (489038-9901-000)

Considerations for evaluating confidence in AI results (i.e. caveats)

Biological samples can be variable and future work could consider filters that would flag and/or remove data that are not likely to be accurately segmented or tracked because of the following features :

- Retraction of small colonies as a result of media change which creates areas of cells that are temporarily bright, rounded, and difficult to segment. As a result, some earlier frames in a time series can return segmentation results that are less accurate, but later time points were well analyzed.
- Debris in the FOV: jumps in tracking that could be due to artifacts like floating debris. We employed stitching vectors that were smoothed across time to avoid this. In some cases where there is significant cell death, inferenced segmentation is impossible because of obscuration of cells in the phase contrast images.

Mean square displacement analysis

The average mean squared displacements (MSD) reported in Figure 4C were calculated as $MSD(d) = \langle [p(t+\Delta t) - p(t)]^2 \rangle_d$, where p is x,y position of the centroid of a detected nuclues, $\Delta t = 1\ h$ is the time lag applied for determining the displacement, d is the distance from the edge of the colony, and the bracket denotes an average over all d. An MSD is determined at all possible $\Delta t = 1\ h$ increments for each cell, and then, MSD values are binned according to distance from the colony edge as indicated in Fig. 4C. We assume the MSD does not depend on time, so we can average over 1 h increments for each cell across the experiment. $\Delta t = 1\ h$ was chosen to reduce the contribution of measurement uncertainty associated with localizing the nuclear object centroid to a single x, y coordinate. In addition to the analysis presented in Figure 4, MSD values were examined for trends as a function of experiment duration and no trend was found. MSD analysis is reported for all tracked nuclear objects from image data in exp0.

References

1. Ling C, Halter M, Plant A, Majurski M, Stinson J, Chalfoun J, editors. Analyzing U-Net Robustness for Single Cell Nucleus Segmentation from Phase Contrast Images. 2020 IEEE/CVF Conference on Computer Vision and Pattern Recognition Workshops (CVPRW); 2020 14-19 June 2020.

2. Blattner, T. et al. A Hybrid CPU-GPU System for Stitching of Large Scale Optical Microscopy Images. *2014 International Conference on Parallel Processing*, 1-9 (2014).
