# Supplementary figures and images for: High-volume, label-free imaging for quantifying single-cell dynamics in induced pluripotent stem cell colonies
Source: PLoS One. 2024 Feb 20;19(2):e0298446. doi: 10.1371/journal.pone.0298446 (PMC10878516; doi:10.1371/journal.pone.0298446)

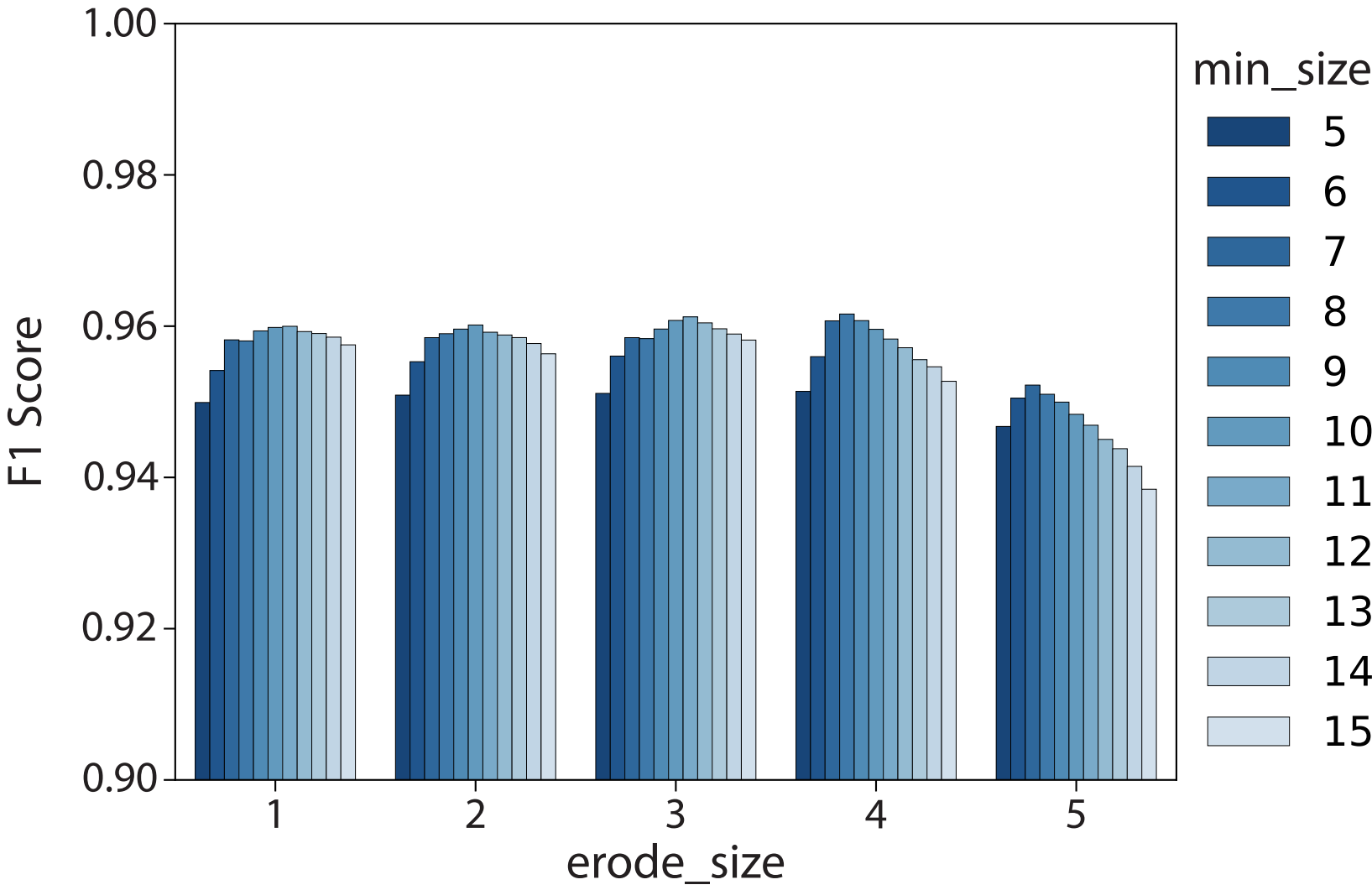

Supplement: S1 Fig — The Fogbank algorithm is applied after the 2D U-Net to separate two or more nuclei that share a boundary and are considered one object. The ‘erode_size’ parameter is varied from 1 to 5 and for each ‘erode_size’ value, the ‘min_size’ parameter is varied from 5 to 15. The highest F1 scores for segmentation accuracy can be obtained with ‘erode-size’ in the range of 1 to 4 and ‘min_size’ in the range of 8 to 10. (PDF) [file pone.0298446.s008.pdf]

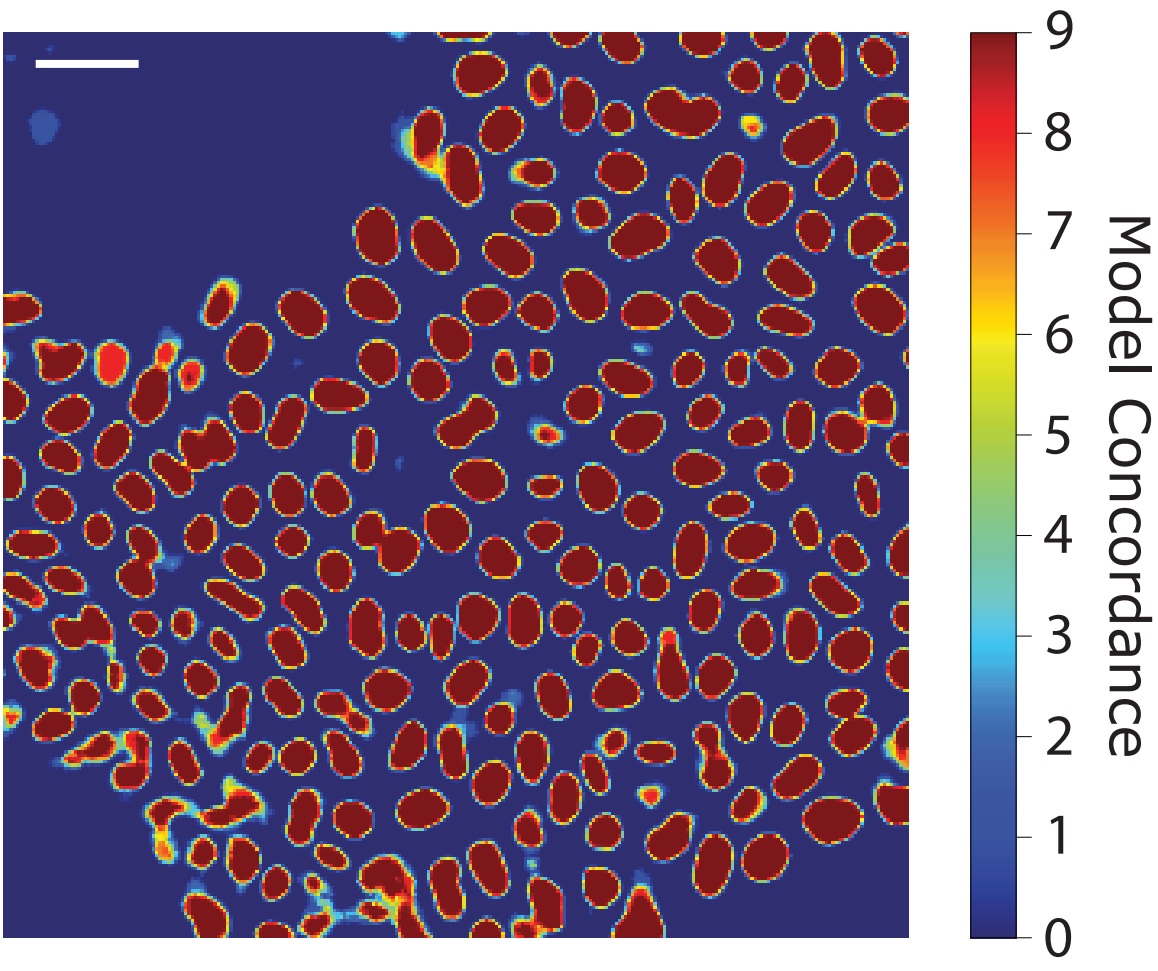

Supplement: S2 Fig — The color scale indicates the number of times a trained U-Net inferred that a pixel was classified as a nucleus. Many pixels exhibit high model concordance (9/9), while other pixels exhibit larger discordance. Scale bar = 25 μm. (PDF) [file pone.0298446.s009.pdf]

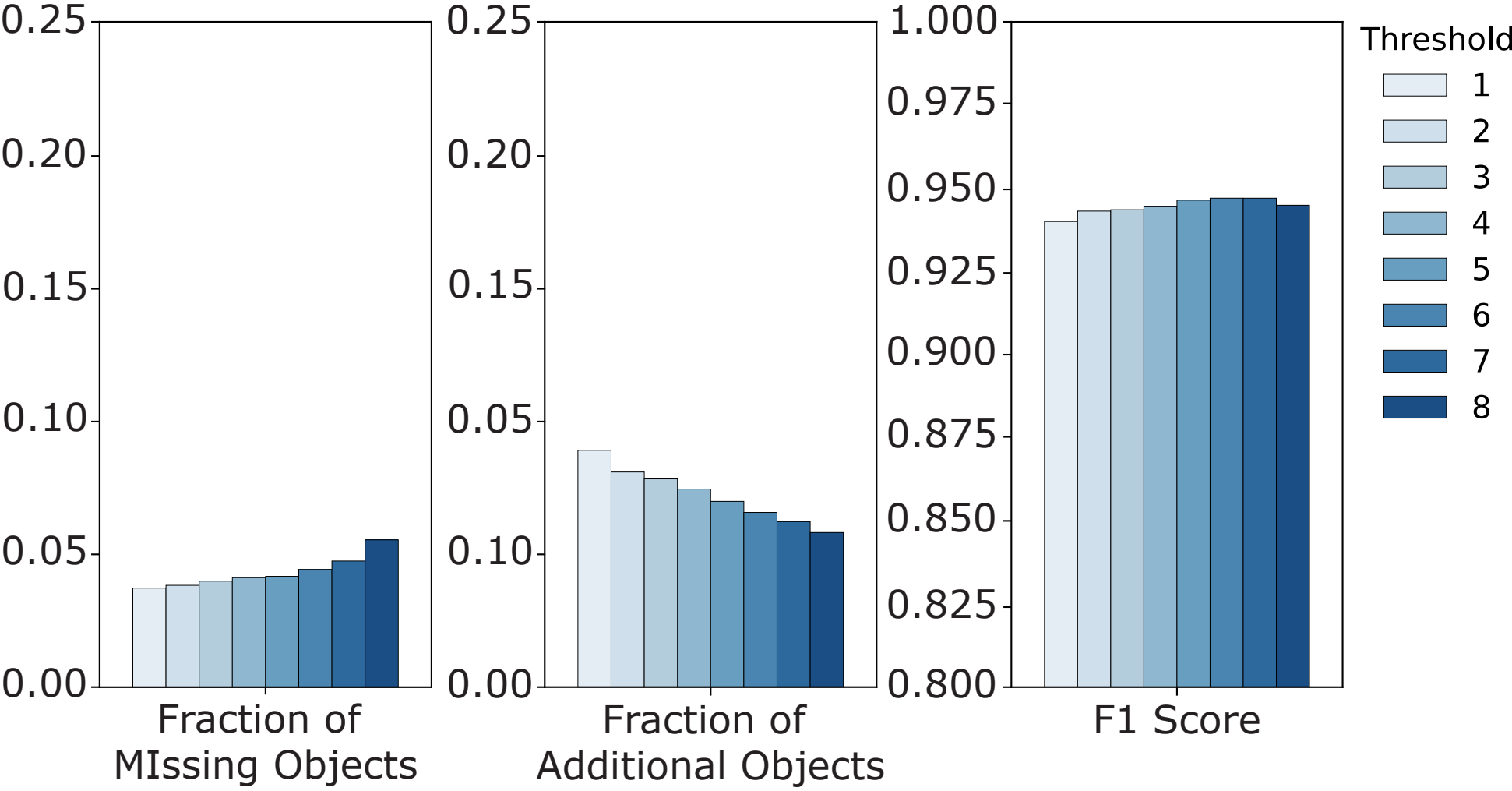

Supplement: S3 Fig — A threshold was applied to the image data in exp0 (a representative sub-image shown in S2 Fig) and the model performance scores: ‘Fraction of Missing Objects’, ‘Fraction of Additional Objects’ and ‘F1 Score’ are plotted as a function of the threshold value. As expected, the ‘Fraction of Missing Objects’ increases with threshold value, the ‘Fraction of Additional Objects’ decreases with threshold value, and the ‘F1 Score’ is highest for intermediate threshold values. (PDF) [file pone.0298446.s010.pdf]

Manual  
GFP  
2D U-Net

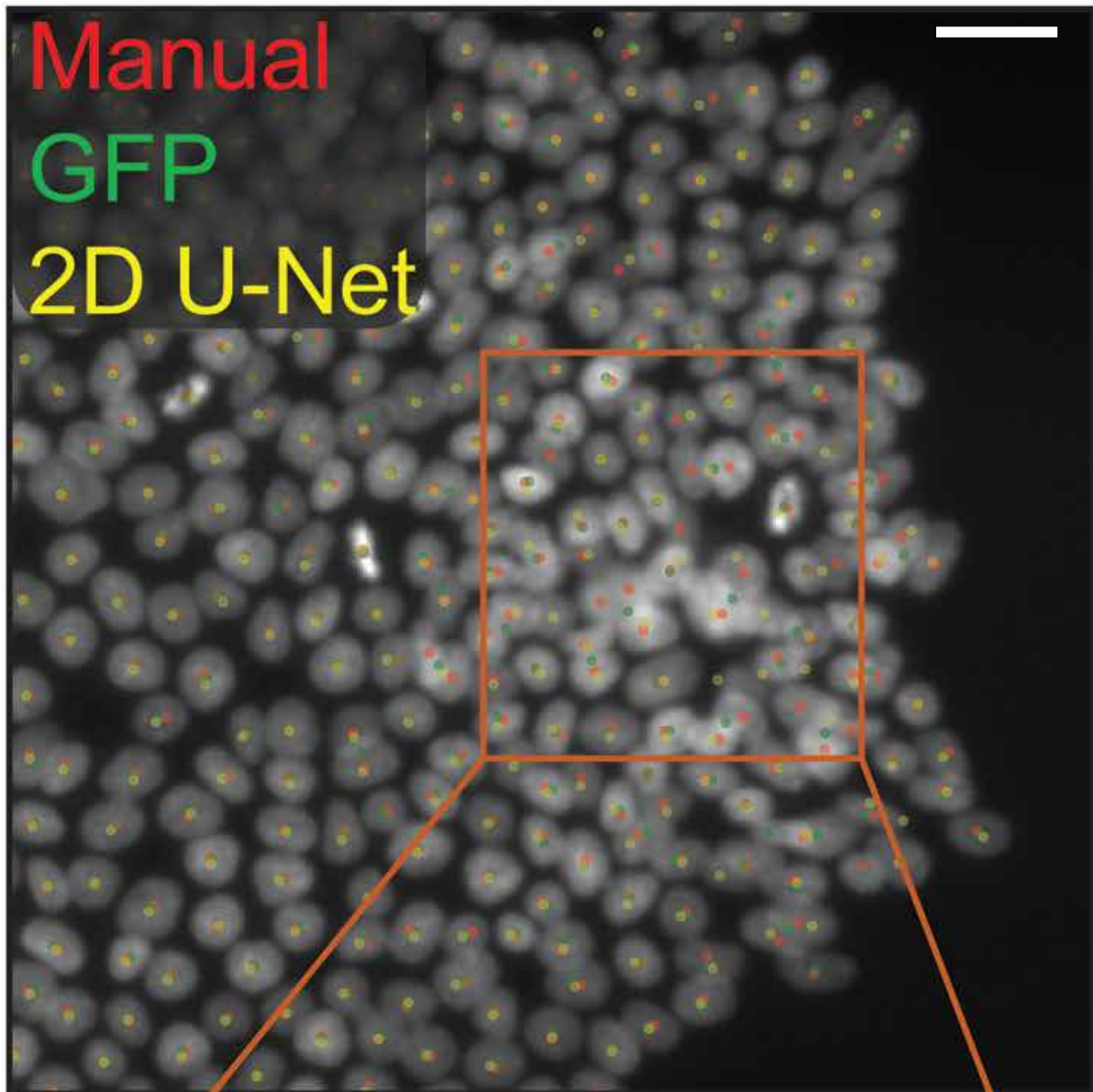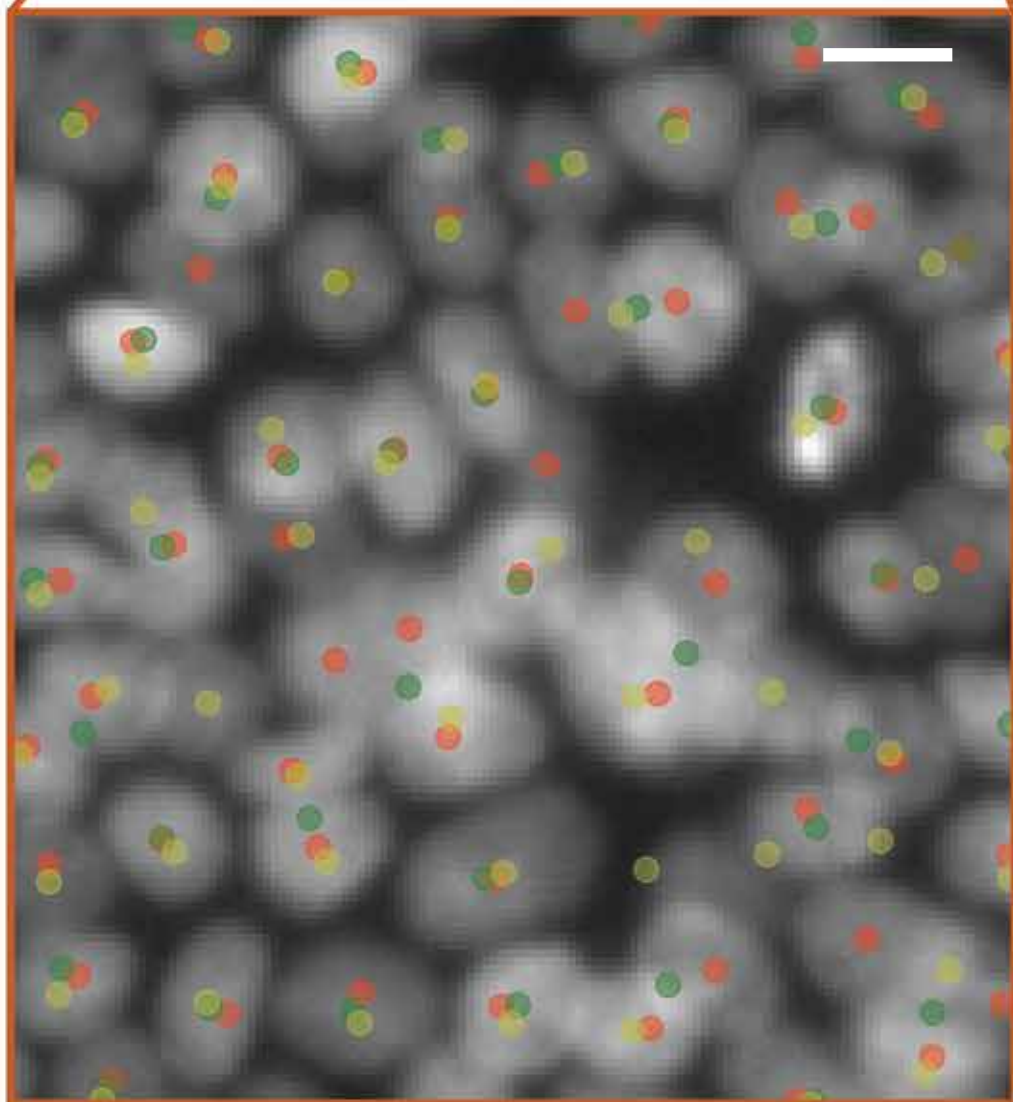

Supplement: S4 Fig — Three sets of detected nuclei data are shown: Nuclei detected by manual inspection of GFP fluorescence images (red dots), nuclei detected by classical image analysis of GFP fluorescence images (green dots) and nuclei detected by AI-based analysis of the phase contrast images (yellow dots). Scale bar = 25 μm. Many image regions illustrated high concordance between the three datasets, whereas the inset square highlighted in orange illustrates a region of high discordance (scale bar = 10 μm). The GFP fluorescence-based automated image analysis tends to merge nuclear objects compared to the manual annotations and the AI-based analysis of the phase contrast images. (PDF) [file pone.0298446.s011.pdf]

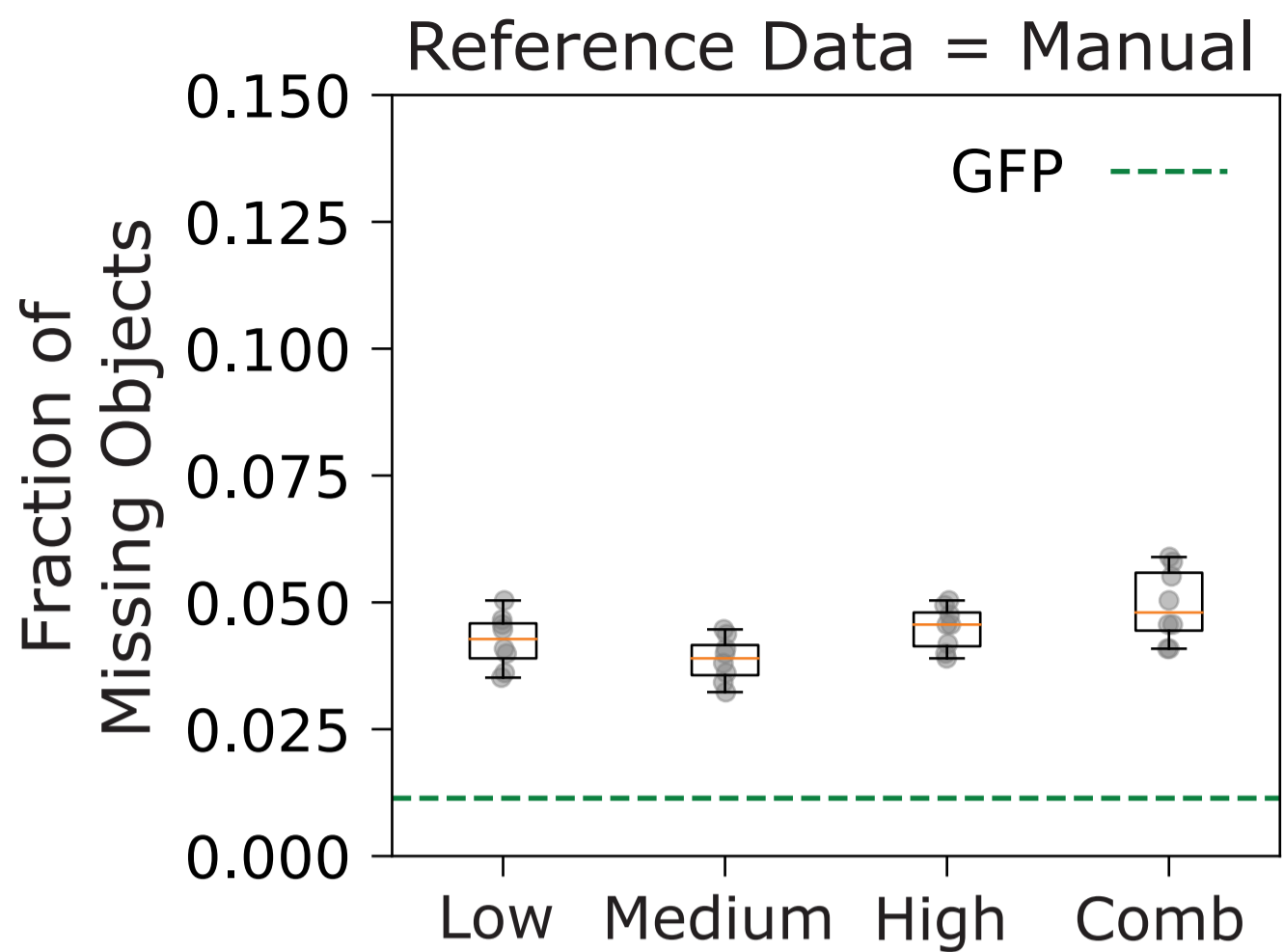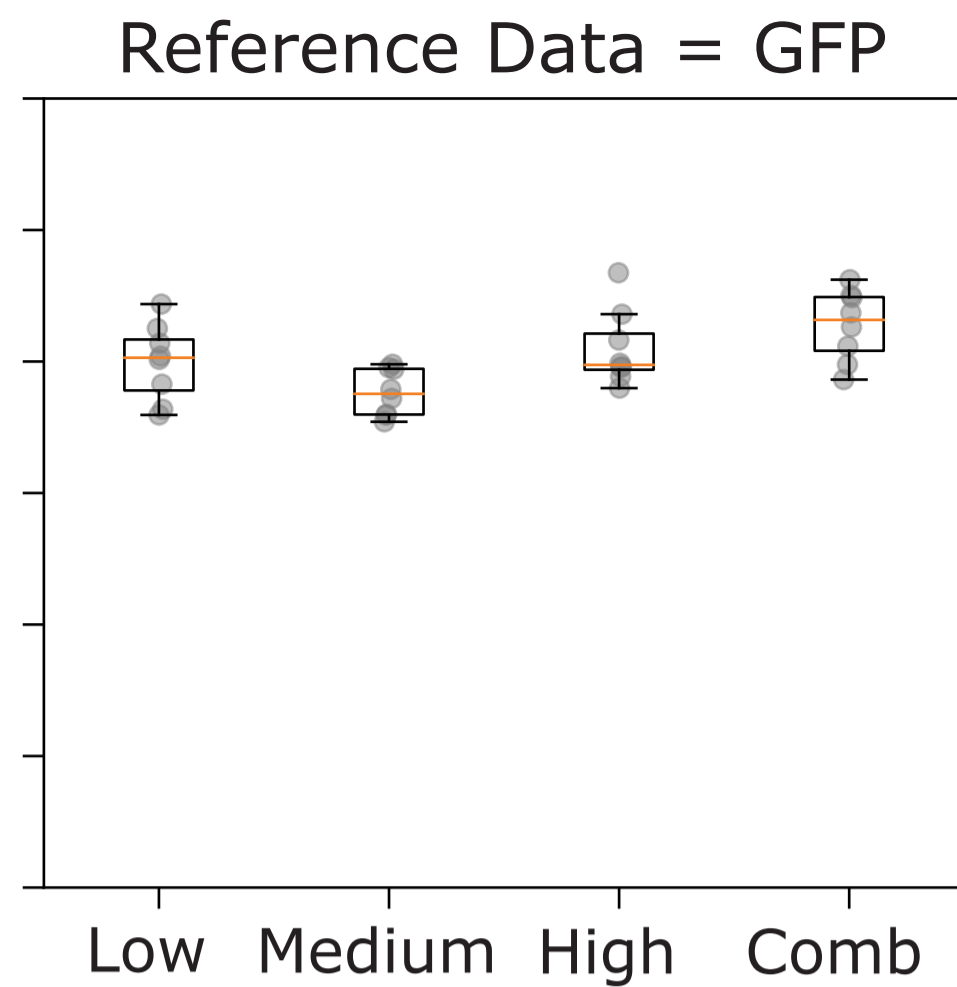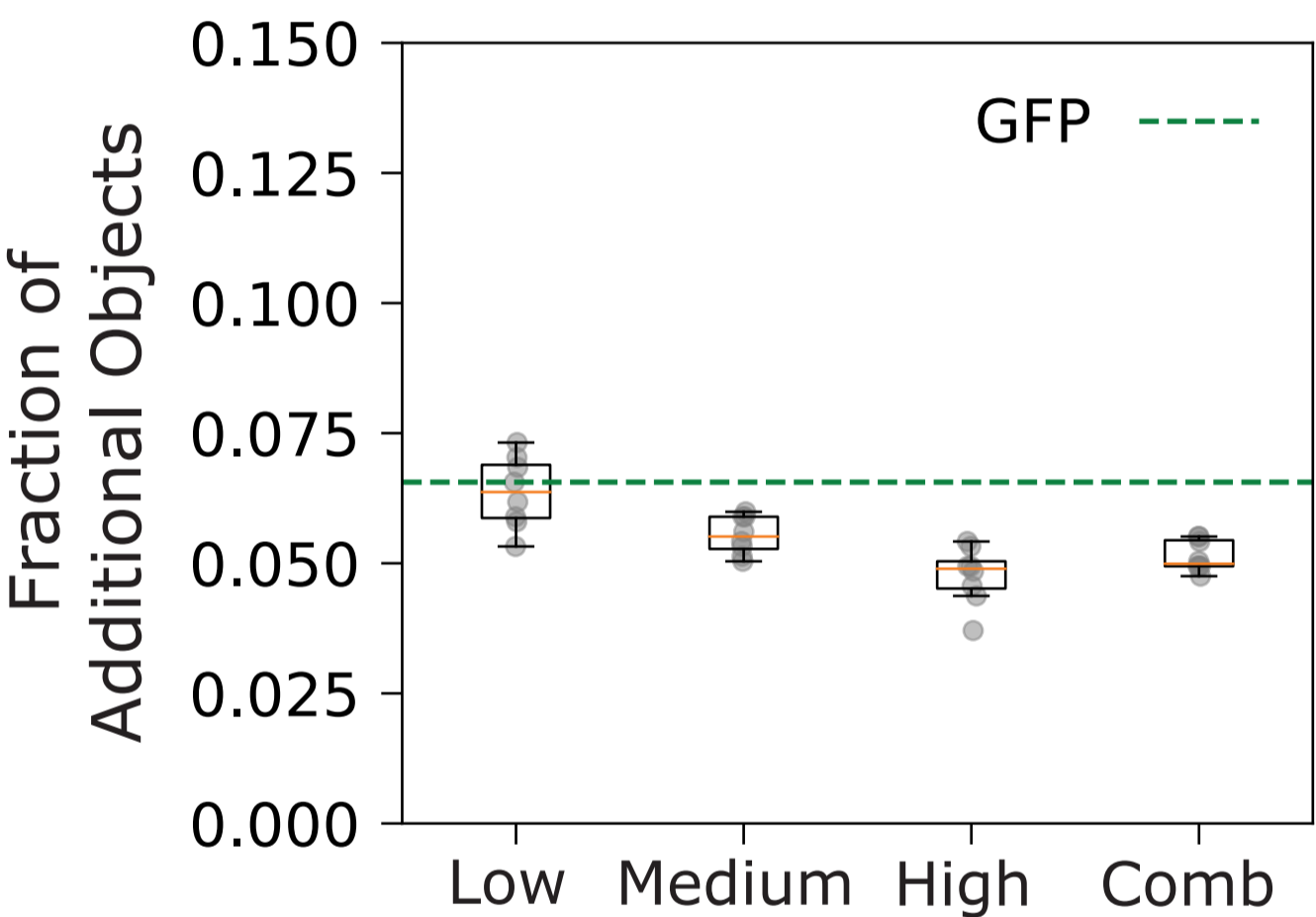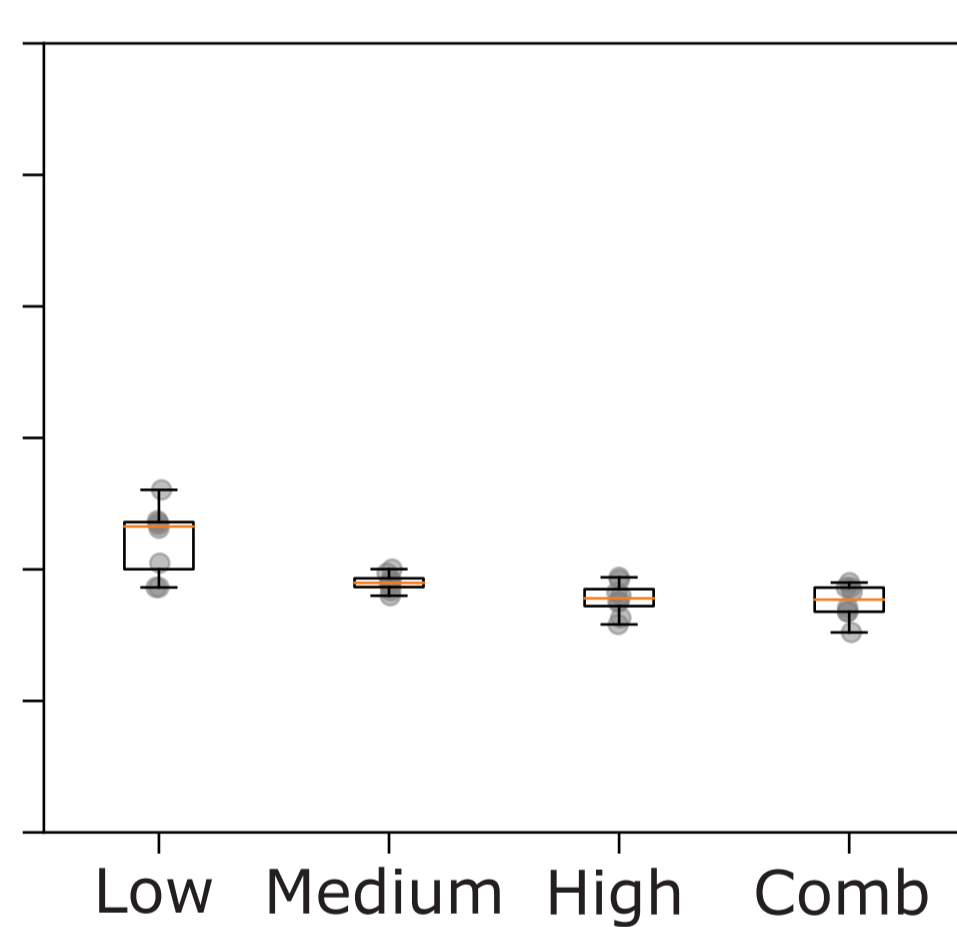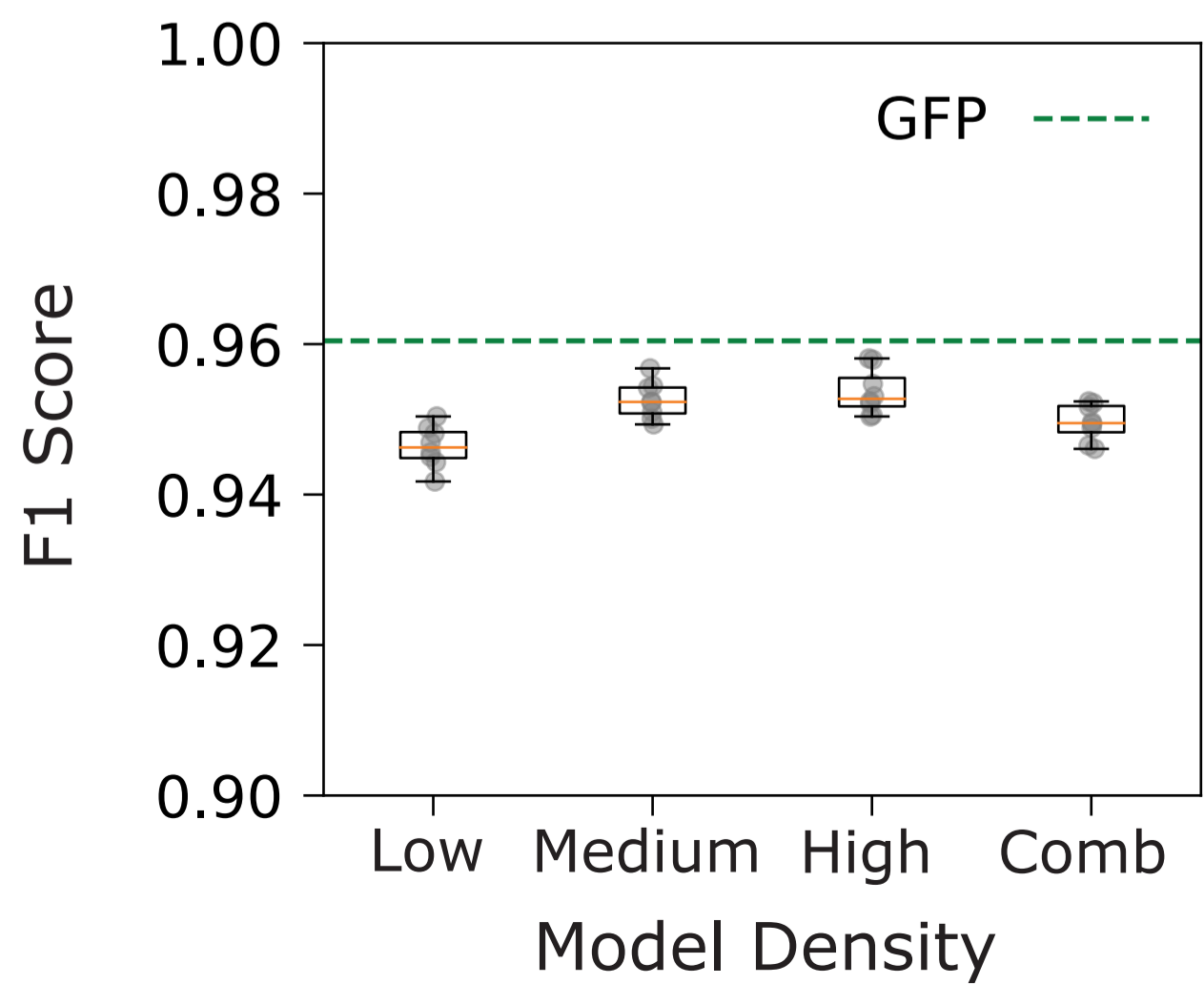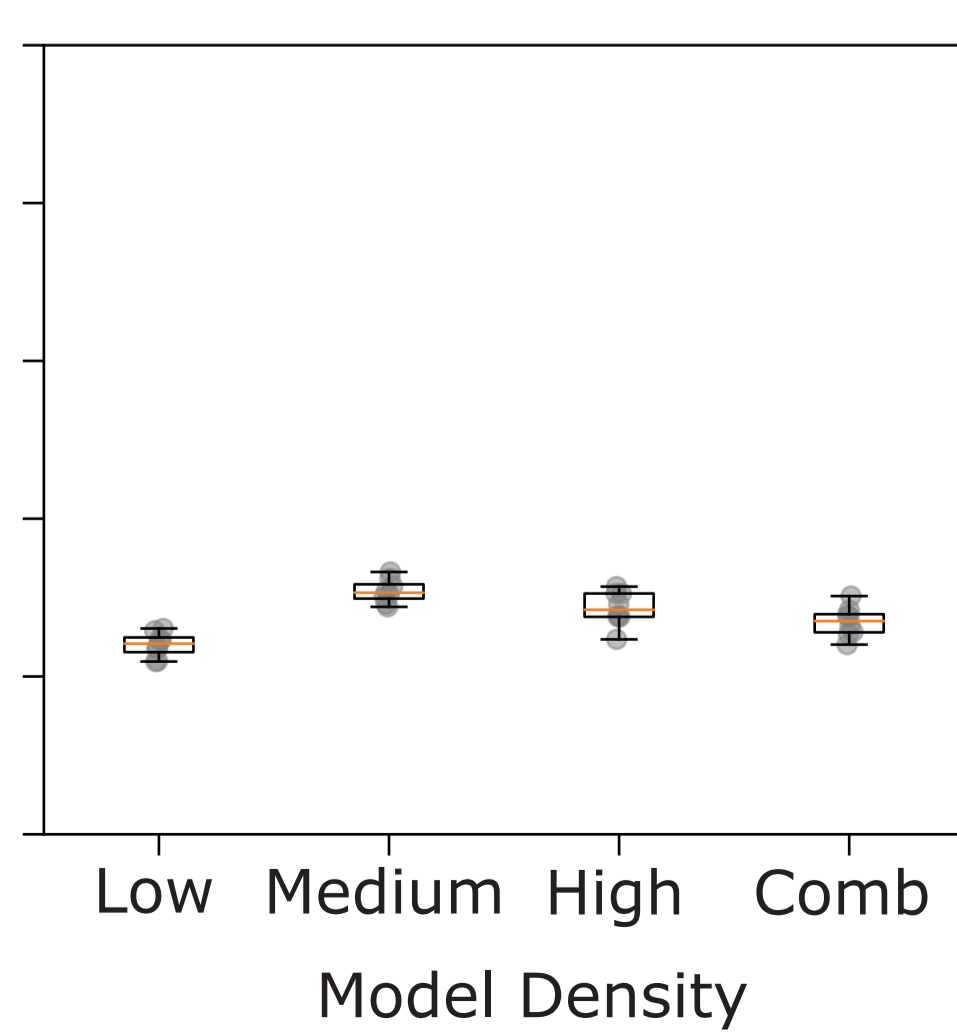

Supplement: S5 Fig — The model performance scores: ‘Fraction of Missing Objects’, ‘Fraction of Additional Objects’ and ‘F1 Score’ are plotted for inferenced data from 4 different U-Net models: the low-cell-density model, the medium-cell-density model, the high-cell-density model and a model trained on a mixture of data from all cell densities. The reference data for computing the scores was derived from either nuclei detected by inspection of the GFP fluorescence images or the nuclei detected by classical image analysis of the GFP fluorescence images. The relative performance of the models is similar using either approach for generating reference nuclei. (PDF) [file pone.0298446.s012.pdf]

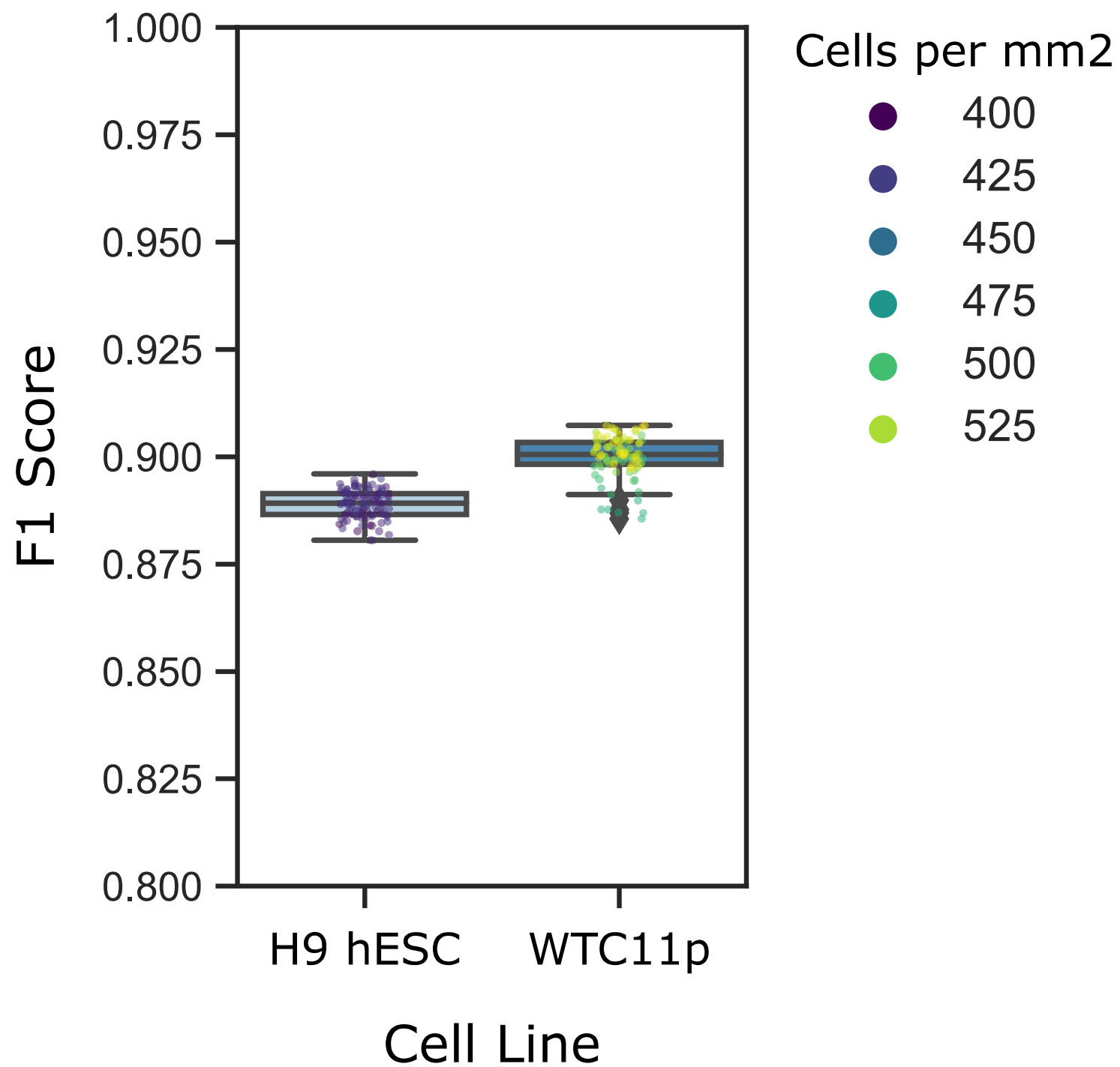

Supplement: S6 Fig — Using the high-cell-density model, the F1 scores are plotted for both H9 hESC (exp6.0) (F1 = 0.89) and the parental WTC11 lines (exp6.1) (F1 = 0.90). Each datapoint represents the corresponding error rate for that frame, and the dot color indicates the density of cells in the frame. Tukey box plots indicate summary statistics for each timelapse dataset. (PDF) [file pone.0298446.s013.pdf]

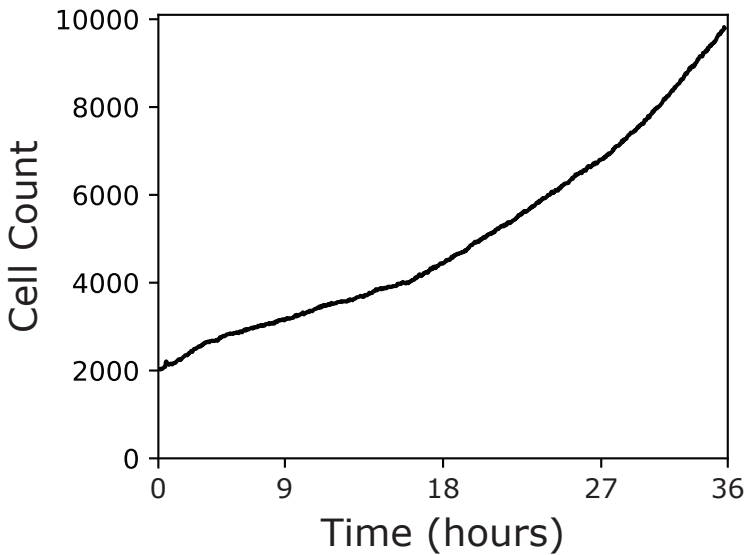

Supplement: S8 Fig — Number of nuclei imaged as shown in S4 Video at each time point during 36 h. (PDF) [file pone.0298446.s015.pdf]

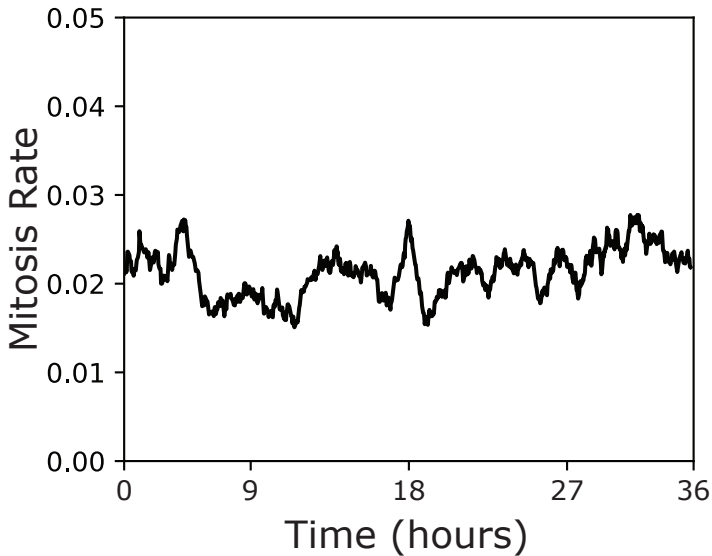

Supplement: S9 Fig — Number of mitotic events that occurred in a 30 min time frame divided by the cell count during that time frame plotted over 36 h. (PDF) [file pone.0298446.s016.pdf]

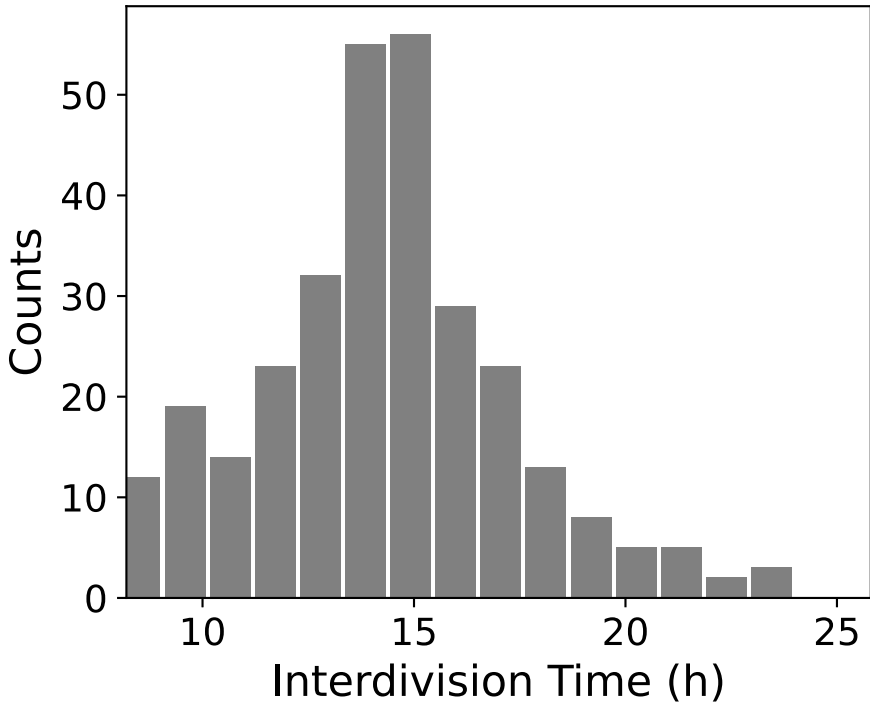

Supplement: S10 Fig — The number of interdivision times (cell lifetimes) determined by tracking of inferenced phase images plotted in 1-hour bins to show the distribution of cell lifetimes (n = 420). (PDF) [file pone.0298446.s017.pdf]
