# Supplementary material for: High-volume, label-free imaging for quantifying single-cell dynamics in induced pluripotent stem cell colonies
Source: PLoS One. 2024 Feb 20;19(2):e0298446. doi: 10.1371/journal.pone.0298446 (PMC10878516; doi:10.1371/journal.pone.0298446)
Supplement: S7 Fig — Green dots represent tracks that have a track length greater than the minimum track length time and are kept. Red dots represent tracks that are filtered out due to having a track length smaller than the minimum track length time. Corresponding phase and fluorescence images are shown for minimum track length times of 0, 1, 2, and 4 hours. Scale bars = 25 μm. (PDF) [file pone.0298446.s014.pdf]

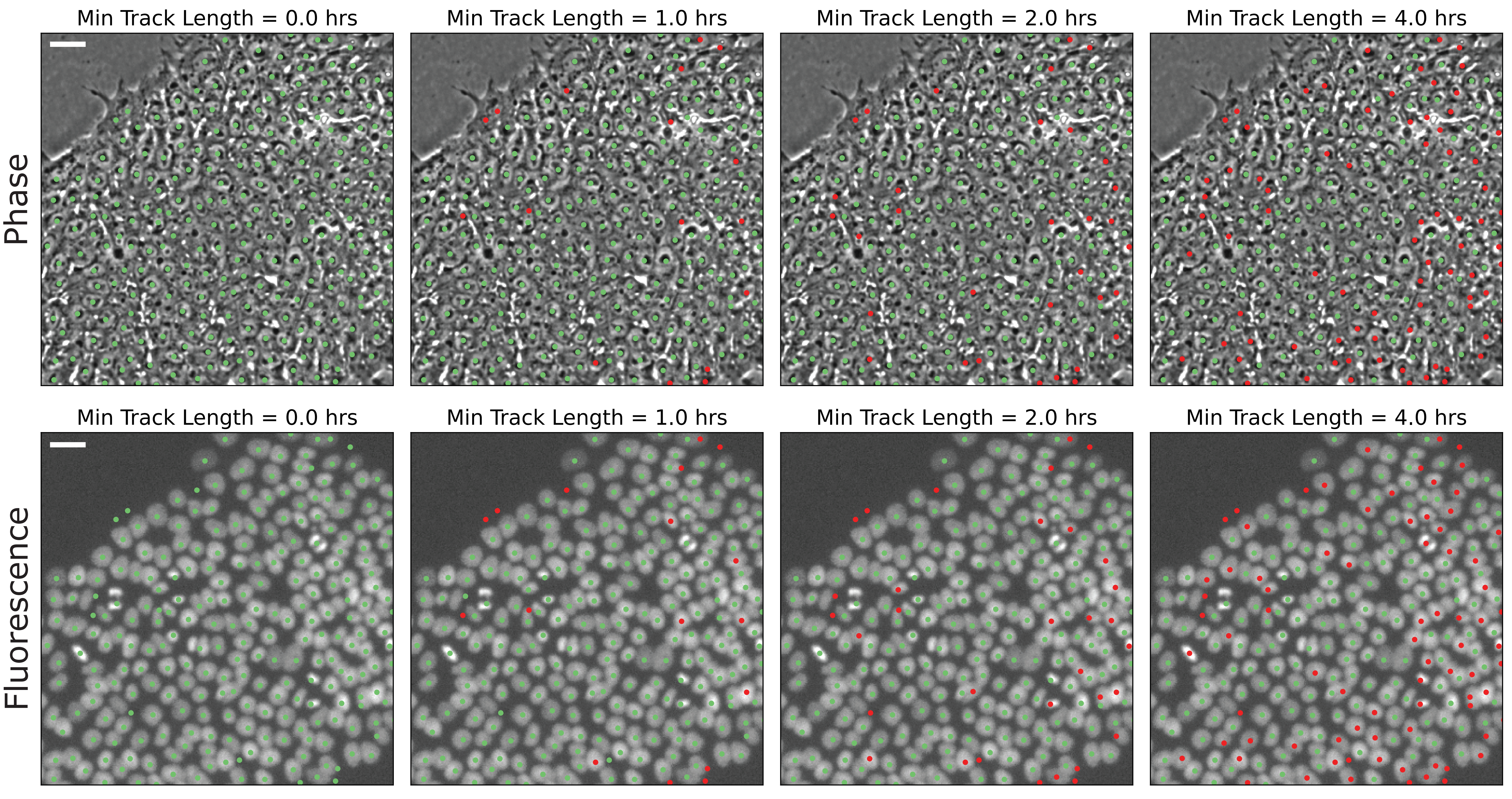

Min Track Length = 0.0 hrs
Min Track Length = 1.0 hrs
Min Track Length = 2.0 hrs
Min Track Length = 4.0 hrs
Phase
Min Track Length = 0.0 hrs
Min Track Length = 1.0 hrs
Min Track Length = 2.0 hrs
Min Track Length = 4.0 hrs
Fluorescence
