# Supplementary material for: High-volume, label-free imaging for quantifying single-cell dynamics in induced pluripotent stem cell colonies
Source: PLoS One. 2024 Feb 20;19(2):e0298446. doi: 10.1371/journal.pone.0298446 (PMC10878516; doi:10.1371/journal.pone.0298446)
Supplement: Attachment — Submitted filename: Response to reviewers.docx [file pone.0298446.s018.docx]

**Response to Reviewers**

We thank the reviewers for their careful reading and thoughtful comments. Below is a detailed response (in red type) to their critique.

Reviewer #1: The authors proposed a new segmentation approach with 2D/3D U-net for human pluripotent stem cells in this study. Using a GFP-labeled nuclear tag, they trained U-Net to detect mitosis. Overall, it was good to demonstrate their algorithm tracking iPSC nuclei for ~20hrs with label-free imaging. However, there are concerns.
The major concerns are (1) novelty, (2) short tracking period, (3) applicability

(1) Recently major microscopies come with AI-based imaging support. Thus, I’m not intrigued by the authors’ approach in terms of novelty or performance. The algorithm is specialized to iPSC culture, does this really outperform others?

In this study we create an analysis pipeline for the routine culturing of iPS cells that includes segmentation, mitosis detection, and tracking, and we explore how a set of sample characteristics and model parameters quantitatively influence performance of the model. We also address reproducibility with respect to day-to-day replicate cell samples and generalizability of the model to other cell lines. This treatment is fairly novel since these are aspects of a new image analysis routine that are rarely reported.

Our primary goal in this study was not to outperform another algorithm, but rather to establish some performance criteria. Having said that, the performance we observe for segmentation is very good (F1 score of approximately 0.94).

Furthermore, the development of an automated method for label-free identification of mitotic cells and tracking daughter cells has, to our knowledge, never before been demonstrated for iPS cells.

Below we address aspects of the novelty of the study, the capability of tracking cells accurately over long times, and the applicability of the model to other pluripotent cell lines.

1) We demonstrate the ability to use single plane phase contrast images to segment and track very large numbers (tens to hundreds of thousands) of individual pluripotent cells per experiment at very high time resolution. While other cell types can be fairly easy to segment, cells such as iPSC, which reside in tightly packed colonies, present particular challenges. In previous studies, very small numbers of pluripotent cells have been tracked with manual segmentation, or automatically tracked using fluorescent markers. By being able to segment and track label-free cells, the image acquisition is noninvasive. Using rapid image acquisition and AI analysis, we demonstrate the ability to quantitatively address each cell with high temporal resolution (every 2 min) over long times (>24 h).
2) Because we interrogate our cells in phase contrast at two-minute intervals, our model allows us to identify and track each cell with high accuracy (frame-to-frame linkage error rate of 0.005).
3) We used automated segmentation of fluorescent nuclei to provide very large training sets for the model (hundreds of thousands of cells). This allows a large variety of cells to be fed into the model, making the model generation efficient and representative of the population. Because we are using single plane data, we can acquire images of hundreds of thousands of cells for training an algorithm in about 10 minutes. The same approach was also used to generate large test sets against which inference results can be compared.
4) With respect to applicability, the development of this analytical pipeline was designed for planar iPSC cultures, which arguably is the most common way of culturing these cells, and therefore is highly applicable to many practitioners. We also demonstrated applicability to two additional pluripotent cell lines which the model was not trained on.
5) The automated detection of mitosis, cell division, and daughter cell tracking of iPSC from phase contrast imaging allows large numbers of multigeneration lineages to be followed in a more accurate and non-perturbing manner; this has not been previously demonstrated to our knowledge.
6) We report the application of a temporal filter to tracked segmented nuclei to increase the confidence in tracked results.

AI-based imaging supports are often proprietary, so we don’t exactly know how they work. There are also open algorithms available. For instance, Bright2nuc was proposed (Atwell, Cell Rep Methods, 2023) to work even in 3D structures. It is important to discuss the advantages of the proposed approach overother approaches, and how it sits in the current perspective.

We thank the reviewer for pointing out this new paper. This study is impressive and similar to our approach in some ways (ie, large training sets), but also different in a number of ways:

- A difference and perhaps an advantage of our method is imaging modality: Atwell et al. used stacks of brightfield images compared to our imaging of single plane phase contrast monolayer cultures, which is what most practitioners currently use and can be acquired and computed with much greater efficiency.
- We demonstrate the direct detection of mitotic events that enables lineage determination and cellular interdivision times, while Atwell et al. do not detect mitosis events or track cell lineage.
- In partial answer to the Reviewer’s next point, Atwell et al. report using TrackMate for continuous tracking of cells for 300 minutes with about a 7 min time interval between frames, and appear not to be able to track any objects for longer than about 11h. They report 50% of nuclei accurately tracked for about 3 h. Our tracking efficiency is approximately 80% of nuclei over 22 h of tracking time, at least in part due to the higher rate of sampling of the cells.
- The AI models are quite different U-Net implementations, most notably in that our 2D U-Net uses a single plane phase contrast image as input, and our 3D U-NET uses a time series of single plane data as input; whereas Atwell et al uses 3D spatial images as input.
- What we have presented and made available is a full cell segmentation and tracking pipeline which includes nuclear segmentation, mitosis detection and daughter cell labeling, and cell and lineage tracking with large scale data.

We now elaborate on some of these points in the Discussion: **bold** type indicates new verbiage.

*The results of this work allow the use of phase contrast images of iPSCs to provide quantitative cell characteristics, such as mitotic rates, cell migration rates and other dynamic behaviors on a cell-by-cell level and with spatial and temporal resolution****. Phase contrast, compared to bright field microscopy,*** ***provides high contrast images from which spatial information can be extracted. The mitotic spindle can be clearly observed in phase contrast images, which are input to a 3D U-Net for mitosis detection.*** ***Data are acquired from single image planes, and therefore can be a more efficient modality compared to bright field imaging.***

And

*In this work, we have acquired and analyzed* ***very*** *large image datasets* ***of hundreds of thousands of cells****, which will be required for uncovering multiparameter correlations in cell populations. Large datasets are also required for the development of reliable models for image analysis, and we demonstrate here the use of fluorescent nuclear probes to automate the process of creating training data. The use of automated segmentation to identify the nuclear foreground from the non-nuclear background allows the creation of very large training and reference data, removing a serious limitation associated with manual annotation.*

And

*The 2D U-Net for nuclear segmentation was trained from single time point images, a process that can be easily* ***and quickly*** *replicated if needed.* ***Our image acquisition system makes it possible to acquire sufficient data for training a model with high cell-density data in about 10 min. Rapid data acquisition also allows us to image many fields of view and many cells in phase contrast at 2 min intervals; this rapid rate helps the accuracy of cell tracking, allowing for a tracking efficiency of approximately 80% of nuclei over 22 h of tracking time. In comparison, Atwell et al. reported 50% of nuclei accurately tracked for about 3 h when acquiring images at 7 min intervals.***

We also now cite this paper in the Introduction section:

***Recently, Atwell et al reported the development of a 3D U-Net to segment individual cells in 3D iPSC cultures imaged in bright field; this model was also trained with large numbers of nuclei (250000) through a combination of manual and model-dependent segmentations.***

(2) The authors tracked cells only 20 to 21hrs, which sounds short to me. I would like to see at least 48 hrs, which should cover two cell divisions. It is interesting that cell proliferation evenly occurs regardless of the location within the colony (Fig 5). If the authors can track cells longer, then the authors address whether the proliferation weans or remains the same throughout the culture period. If it can apply for much longer, the approach can be applied for differentiation studies, too.

Segmentation and tracking with the algorithm are not dependent on time. Cells can, of course, be imaged for longer, depending on the logistical details associated with cell density and medium exchange. To demonstrate this, we have added a 36 h imaging series to the posted data and Supplemental Information. This is discussed in the text at the end of discussion of Figure 4, and is shown in Supplemental Figures 8, 9, 10 and Supplemental Video 4.

(3) Establishing an algorithm is important, and it is more important that the algorithm can be applied to any given system/image. Is it possible? It should be at least discussed, and if possible, please demonstrate if the algorithm works on images from different microscopes/different imaging settings.

We have shown that the model works well for two cell lines in addition to the cell line that it was trained on, the parental iPSC line from which the H2B-GFP line was made, and a hESC line (see paragraph starting with line 377 and the data shown in S6 Fig). We now emphasize the differences between these cell lines and the cell line that was used for training. We also additionally tested the model on image data acquired on two different microscope systems; this is now also discussed in that paragraph: *To test the robustness of the segmentation and tracking model for different colony-forming cell lines, we applied the model to phase contrast images from the parental WTC-11 iPSC line and the embryonic stem cell line H9****. These two cell lines are distinct from the cell line that was used for training the algorithm. The GFP-producing cell line used in training was derived from the WTC-11 line, but subtle differences in derived lines are often observed. We expected that the embryonic H9 cell line might be very different from the iPSC cells used for training. Again, subtle differences in cell and colony morphology seem apparent by eye. The scores for performance of the model when applied to these two cell lines were determined by comparing inferenced results with automated segmentation of reference data created by staining these cells with Spy595-DNA (see Methods). Model performance for these cell lines is similar to what was observed with the GFP-producing cell line, indicating model generalizability to these other pluripotent stem cell lines (data shown in S6 Fig). In addition, we compared F1 scores on data taken from 2 nominally different microscope systems and found negligible differences (F1-scores of 0.95 and 0.94; see Supplemental Materials for details).***

Finally, U-Nets are often adapted or trained for specific image datasets or image analysis problems. What constitutes sufficiently similar image characteristics to confirm that any model is applicable to any other dataset has not been systematically demonstrated. To highlight our contribution to rapid training and testing of a U-NET for solving an analysis problem, we have added to the Discussion: ***We have developed an image acquisition and analysis pipeline that can be efficiently trained and inferenced on a new dataset. Hundreds of thousands of cells for training are imaged in 10 min, and training and inferencing takes a few hours.***

(4) In Figure 6, the authors demonstrate that fluorescence excitation reduced cell number due not to reduced proliferation but to increased apoptosis. It wasn’t unclear if the authors were able to detect such cells in this approach. Can it be dictated by the likes of the drop-out rate? Is it only detectable with manual inspection (line 537)?

We did not directly quantify cell death, but the small number of cells relative to the control (as shown in Figure 6A) suggested that cell death was occurring, and the presence of debris supported that conclusion (see S5 Video that illustrates the appearance of dying cells within the colonies).

(5) Something that I couldn’t get was a “fraction of additional objects”. What actually are the “additional objects”? In addition, in Figure, it was labeled as “extra objects”.

We employed the term “fraction of additional objects” in place of the term “false positives” as stated in Methods under *Assessing the accuracy of label-free segmentation*. “*The number of false positives or over-segmented objects are normalized by the reference cell count and reported as fraction of additional objects, and the number of false negatives or under-segmented objects are normalized and reported as the fraction of missing objects.”* This terminology is consistent with the paper cited (Caicedo, J.C., Roth, J., Goodman, A., Becker, T., Karhohs, K.W., Broisin, M., Molnar, C., McQuin, C., Singh, S., Theis, F.J. and Carpenter, A.E. (2019), Evaluation of Deep Learning Strategies for Nucleus Segmentation in Fluorescence Images. Cytometry, 95: 952-965. <https://doi.org/10.1002/cyto.a.23863>).

However, we were inconsistent with nomenclature in the legend for Figure 2. Thanks to the Reviewer for pointing that out. That is now corrected.

(6) The figures mainly displayed the training and confirmation process. I would like to see more demonstrations of what the algorithm can do/apply.

The overarching goal of this paper is to describe and characterize the development and performance of a novel quantitative imaging and image analysis pipeline for quantifying and tracking individual label-free pluripotent cells and their progeny in 2D colonies.

In addition, we show two Figures that demonstrate the power of the pipeline.

In Figure 5, we show that this analysis allows determination of the effect of position in the colony on migration of cells and on rate of mitosis.  These data were based on analysis of approximately 10,000 cells, and that fact is now added to the Figure legend. We have now also added this conclusion to the Results in order to make it more apparent that the results are significant:

***While this may not be a surprising result, the large number of cells that are included in this analysis (approximately 10,000) provides confidence in this conclusion. Average mitotic rates, on the other hand, do not appear to depend on distance from the colony edge (Fig 5D), indicating that while cells are more migratory near the edges of the cells, they are not dividing at a higher frequency.***

In Figure 6, we demonstrate that when exposed to damaging levels of excitation light, the number of cells decrease, suggesting either cell death was occurring, or cell division was curtailed. But the ability to independently measure mitosis rates shows that the remaining cells demonstrate a normal mitosis rate. This observation is significant because it indicates that response of the cells to damaging levels of light is not homogeneous across the population and suggests that the mechanism of phototoxicity is more nuanced.

In order to emphasize the significance of these results we have added more explanation in Discussion.

*In addition, access to spatial information of individual cells permits the assessment of the effects of changes in culture conditions on cell-cell interactions, the relationship of location within the colony to cell division and motion.* ***For example, these data, made possible by the spatial and temporal capabilities of the*** ***quantitative imaging and image analysis pipeline, suggest that migration rate and mitosis are not correlated, and that cells do not divide any more frequently if they reside at the edge of a colony than if they are in the middle of a colony.*** ***The large number of cells in this analysis provides confidence in this result. We also demonstrated the surprising observation of photo-induced toxicity of some cells in a population, while the mitosis rates in other cells appeared to be normal.***

To address the Reviewer’s comment directly, we also added the following data:

We have added data from a 36 h tracking sequence to the Supplemental info and calculated the distribution of interdivision times within the population, as well as cell count and mitosis rate over time; these results are discussed in Results and shown in S4 Video and S8-S10 Figures (see response to (2) above).

Reviewer #2: High-Volume, Label-Free Imaging for Quantitative Single-cell Dynamics in Induced Pluripotent Stem Cell Colonies
• Overall Thoughts
o In this manuscript, the authors demonstrate a machine-learning method for nuclear segmentation, cell tracking and mitosis detection in phase contrast (PC) imaging of iSPC colonies. Due to the toxicity of prolonged fluorescent imaging, such a workflow would allow for long-term quantification of cell state and dynamics over much longer periods than otherwise feasible. Additionally, the issue of photobleaching often impacts experimental design, irrespective of cell health, limiting the scope in some instances. Circumventing these issues, at least in this specific context, would present a significant step forward in microscopy experimental design. Indeed, the capacity for AI to help decrease the amount of illumination necessary in experiments remains one of the largely untouched areas in microscopy. This work offers a step in this direction. Moreover, by developing a model which considers the information contained in PC images, this work points towards a class of models built upon this dye-free ground truth.
o Their proposed algorithm takes the form of linked 2D and 3D U-Nets trained on fluorescent data for nuclear segmentation. The authors evaluate the accuracy of their model by testing it at multiple cell densities and cell lines, suggesting the model is plausibly generalizable. Moving forward, it will be interesting to see whether such a workflow functions on other cell types and in other imaging contexts. Further, they use this model to offer preliminary quantification of cell motility and more importantly, the mal effects of fluorescent imaging on colony health, suggesting this form of analysis can elucidate the details of this toxicity. The authors also assert they envision this pipeline facilitating the study of gene expression in a label-free environment.
o In total, this well-written manuscript presents an imminently useful pipeline for tracking and quantifying iPSC colony behavior dye-free.
• Review Criteria
o Originality
♣ This work builds on the authors’ previous models by combining and optimizing pieces of other pipelines into a cohesive workflow for segmentation, tracking and cell state determination. As far as can be discerned, this work does not appear in any other publication.
o Technical Standards
♣ The statistical significance of quantified results is effectively measured and communicated. In the more qualitative sections, the authors clearly describe differences and show, to the extent possible, their reasoning through their data.
• The one instance I would like to see extended and more robust reasoning is the assertion that the pipeline functions best at the highest colony density, as explained below.
o Interpretation
♣ The data present clear behaviors from which the authors derive reasonable and justified conclusions.
o Presentation
♣ The manuscript is well-written and structured.
o Ethics
♣ No issues are readily apparent.
o Data availability
♣ The data and code are all available through the stated repository, and the code itself is sufficiently documented.
• Specific Comments
o Throughout, the authors indicate (and the data demonstrate) that the pipeline’s accuracy is best when applied to the highest density colonies. I would appreciate and suggest a discussion of the authors’ interpretation and intuition regarding this fact. It is entirely possible there is a biological reason that I am missing, but if there is an algorithmically relevant reason, that could be discussed. Additionally, there does not seem to be the same trend in accuracy as a function of density when plotted in Fig 2C. If anything, the F1 score decreases as the density increases. I would suggest the authors elaborate on these ideas.

We agree with the Reviewer. We do not intend to claim a large difference in performance of segmentation models (indicated by the F1 score and other metrics) that were trained on different densities of cells. With respect to segmentation, we now state in Results (changes shown here in bold face): … *the models perform similarly over the entire 20 h experiment,* ***whether*** *~~when~~ cells are at relatively low densities* ***or if*** *~~and when~~ cells are at relatively high densities. Comparison of the fraction of additional objects, the fraction of missing objects and the F1 score between 2D U-Net models showed that the performance of the* ***3 segmentation*** *models was not highly sensitive to cell density, and the* ***high-cell-density model*** *performed nominally better* *than the others… While the difference in performance of the 3 segmentation models* ***is negligible, a distinction becomes apparent*** *when cells are tracked over long times…*

We observe that small differences in segmentation accuracy are additive and are apparent when tracking over long times (i.e., many frames; see Figure 4E). The subtle difference in linkage error rate (Figure 4C) creates the discrepancies in track lengths seen in 4E for the models trained on the different cell densities. The GFP labeled cells have a linkage error rate of approximately zero, but the plot indicates that not all cells experience a 22 h track length. This could be due to attrition in cell number due to cell death, lost tracks, the presence of debris, or movement of cells out of the field of view. We now clarify these points in the Results associated with Figure 4, and have added the following:

***It is interesting to note that all models performed similarly with respect to segmentation over a wide range of cell densities as shown in Figure 2C; i.e., regardless of the cell density that the models were trained on, they performed similarly at low and high densities of cells. One difference between the 3 models was the number of cells that were provided for training. While the total number of pixels used for training the 3 models was kept constant, the number of cell objects*** ***in the training sets were 38875, 80997, 214944 for the low-cell-density, medium-cell-density and high-cell-density* *models, respectively, suggesting that the U-Nets are sensitive to number of objects used in training.***

We have also edited text to make it very clear that the models were trained on images of cells at 3 different cell seeding densities. The text in Results under Evaluation of 2D U-Nets now reads:

*To* ***increase the likelihood of*** *producing a model that is applicable for different datasets and over the times that cells are in culture, we trained* ***three*** *models* ***with images from three different wells of cells that were prepared by seeding each well with a different number of cells.*** ***We refer to these 3 models as low-cell-density, medium-cell-density, and high-cell-density models.*** *The models were tested against images of cells at different densities of cells to evaluate the generalizability of the models for different cellular contexts.*

o In Fig4, the authors show the effect of track length filtering on model accuracy. As the minimal track length increases, the number of spurious detections decreases, but the number of missed detections increases. This suggests some ideal track length—indeed the plot of F1 score as a function of minimal track length seems to display a rough peak of sorts. I would be interested in the authors’ thoughts on whether such an ideal threshold exists, and if so, what that might mean physically.

Indeed, there is a trade-off. Depending upon the experiment and the question being addressed, one might want a high statistical sampling and can tolerate some error; but for some questions, you might want the highest confidence and are willing to reduce the number of cells sampled. This is now more fully elaborated on in the Discussion section: ***The use of a temporal filter to eliminate shorter tracks can be applied selectively depending on the experimental objectives. For example, if a large statistical sampling was desired for determining the rate of population expansion, one might use a less stringent filter than if the long-time fluctuation in cellular expression of a gene reporter were of interest.***

♣ Additionally, while the data do suggest that pipeline functions best at highest colony density, this conclusion would be strengthened by a clear singular measurement of this fact. For example, if the authors could quantify by how much better the pipeline performs, that could be useful.

To be clear, we have compared three different models, where each model was trained on cells seeded at three different densities, and we have made that clearer in the text [see above]. By using GFP fluorescence as reference for accurate cell tracking, we can compare the percentage of cells that are as accurately tracked at 20 h for the 3 models. Those values (as a percentage relative to GFP tracks) are:

High-cell-density model: 79%

Medium-cell-density model: 52%

Low-cell-density model: 42%

These values are now reported in the Results section.

o Fig5 represents what I consider to be the manuscript’s weak link. I appreciate and respect that the authors want to demonstrate the pipeline’s usefulness in answering physical questions. In that vein, these data are sufficient. However, this section reads as rather thin when compared to the totality of the work. In other words, the main conclusion from this figure—that cells move more at the colony periphery than the center—is so unsurprising it falls flat as a demonstration of the algorithm’s use.

♣ Instead, I would move Fig6 before this one. Highlighting the issue of phototoxicity and how this pipeline serves to mitigate it strikes me as much more impactful than the result(s) of Fig5. In fact, I would suggest the authors bring in this idea to Fig5, possibly in showing the effect of illumination on cell motility. This would both demonstrate that the pipeline facilitates such measurements, but also connects it to what has come before.

We appreciate the Reviewer’s discussion and concern.

Respectfully, however, we disagree with the Reviewer on this point. While the result we report might have been expected, it is a conclusion that can been largely anecdotal. The capability that we have shown for quantifying such characteristics allows for objective comparisons to be made between different iPS cell cultures within and between laboratories. In Figure 5 we show that this analysis allows a confident determination of the effect of position in the colony on migration of cells and on the rate of mitosis simultaneously. While cells are more migratory near the edges of the colonies, they are not dividing at a higher frequency, suggesting that migration rate and mitosis are not correlated, and that cells do not divide any more frequently if they reside at the edge of a colony than if they are in the middle of a colony. These data were based on analysis of 10,000 cells. We think this correlation, particularly over the large number of cells, is significant.

We have added the following to the Discussion section to make clearer the significance of the findings reported:

*In addition, access to spatial information of individual cells permits the assessment of the effects of changes in culture conditions on cell-cell interactions, the relationship of location within the colony to cell division and motion.* ***For example, these data, made possible by the spatial and temporal capabilities of the*** ***quantitative imaging and image analysis pipeline, suggest that migration rate and mitosis are not correlated, and that cells do not divide any more frequently if they reside at the edge of a colony than if they are in the middle of a colony. The large number of cells in this analysis provides confidence in this result. We also demonstrated the surprising observation of photo-induced toxicity of some cells in a population, while the mitosis rates in other cells appeared to be normal.***

In addition, we have added another additional finding:

The distribution of over 400 interdivision times across the population that were calculated from a 36 h tracking sequence; these findings plus the average mitosis rate and the change in cell count over this time frame, are discussed in Results and shown in S8-S10 Fig.

o There are a few minor elements of Fig6 I would suggest that the authors change. The titles of the plots in A and B do not effectively communicate that they represent different illumination amounts. I would make this clear to the reader. Similarly, this applies to the individual boxes in the turkey box plots as well.

We thank the reviewer for pointing that out. We have modified Figure 6 to make it more clear by adding “Excitation Light” or “Excitation” with the 1x, 1.4x, 2.1x, and 3.6x labels.

o Otherwise, outside a relatively small number of minor proofreading errors, the manuscript is well-written and structured.
